# Use of helminth therapy for management of ulcerative colitis and Crohn's disease: a systematic review

**DOI:** 10.1017/S0031182021001670

**Published:** 2022-02

**Authors:** Victoria Emma Shields, Jan Cooper

**Affiliations:** Warwick Medical School, University of Warwick, Coventry CV4 7AL, UK

**Keywords:** Crohn's disease, helminth therapy, immunomodulation, inflammatory bowel disease, parasites, ulcerative colitis

## Abstract

The incidence rate of inflammatory bowel diseases is increasing in developed countries. As such there is an increasing demand for new therapies. The aim of this systematic review was to investigate whether there is evidence to support the use of helminth therapy for the management of Crohn's disease and ulcerative colitis. Four databases (PubMed, Embase, Medline and the Cochrane Central Register of Control Trials) were searched for primary evidence in the form of clinical studies. Nine studies were suitable for inclusion: five double-blind randomized control trials and four open-label studies. This review divided the results of the studies into two categories: (a) the efficacy of helminth therapy and (b) the safety of helminth therapy. Results regarding the efficacy were mixed and a conclusive answer could not be reached, as there was not enough evidence to rule out a placebo effect. More research is needed, particularly studies with control groups to address the possibility of a placebo effect. Despite this, all nine studies concluded helminth therapy was safe and tolerable, and therefore there is currently no evidence against further exploration of this treatment option.

## Graphical abstract

**Figure:**
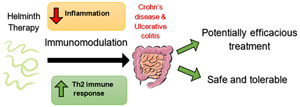

## Introduction

Inflammatory bowel disease (IBD) is a group of chronic inflammatory disorders that affect the gastrointestinal tract, characterized by relapsing and remitting episodes of inflammation resulting from an uncontrolled immune-mediated inflammatory response (Malik, 2015; Shapiro *et al*., 2016). Crohn's disease (CD) and ulcerative colitis (UC) are the two main types of IBD seen in populations (Fakhoury *et al*., 2014). The cause of IBD is currently unknown, however there has been evidence suggesting that genetic and environmental factors may be linked to its development (Baumgart and Carding, 2007).

The incidence of immune-related diseases such as IBD is rising in developing countries. Between 1990 and 2017 the number of people with IBD globally increased from 3.7 million to more than 6.8 million (95% UI 6.4–7.3) – an increase of approximately 83.9% over 27 years and this trend is projected to continue (GBD, 2017 Inflammatory Bowel Disease Collaborators, 2020). The hygiene hypothesis (Strachan, 1989) suggests that this could be due to medical advancements and lifestyle changes reducing our exposure to infections that would have previously been common, and that a link exists between exposure to these infections and modulation of our immune system (Okada *et al*., 2010).

Currently, there is no known medical cure for IBD and therefore there is interest in exploring new avenues of treatment (Fakhoury *et al*., 2014). Treatments offered at present are limited to medications to control the symptoms of the disease and reduce inflammation, or surgery to remove affected sections of the bowel. Unfortunately, many of the medications cause adverse effects, especially when required at higher doses (Fakhoury *et al*., 2014). Surgical resection of the bowel can sometimes resolve UC if the entire affected section of the bowel is removed and there are no extra-intestinal symptoms. In CD this is not a curative option and is usually used to manage disease complications (Baumgart and Sandborn, 2007).

Helminths have co-existed with humans for millions of years and have adapted mechanisms to modulate their host's immune system to support their own survival (Helmby, 2015). Studies in animal models have produced evidence demonstrating the ability of helminths to suppress inflammation (Smallwood *et al*., 2017; White *et al*., 2020; Lothstein and Gause, 2021). In animals with helminth infections a polarization towards a Th2 immune response which has a role in suppressing inflammation was identified. Recent research has linked this to the release of damage-associated molecular patterns (DAMPs), which include trefoil factor 2, ATP and chitinase-like proteins, which are released when a helminth invades epithelial barriers (Gause *et al*., 2020). This leads to an increase in regulatory T cells and certain cytokines, including IL-4, IL-5, IL-10, IL-13 and TGF-*β* (Ayelign *et al*., 2020; Maizels, 2020; Lothstein and Gause, 2021) and a decrease in pro-inflammatory cytokines such as IFN*γ* and TNF (Smallwood *et al*., 2017; Ayelign *et al*., 2020). There is also evidence that helminth infection can suppress the Th1 and Th17 immune responses which have a crucial role in the pathogenesis of autoimmune diseases (Walsh *et al*., 2009; Lothstein and Gause, 2021). Therefore, there may be potential for the use of helminths to improve symptoms of immune-related disorders such as IBD (Maruszewska-Cheruiyot *et al*., 2018).

A previous systematic review looking at the use of helminth therapy for inducing remission of IBD only identified two studies that were suitable for the review, and concluded that more evidence was needed (Garg *et al*., 2014). This systematic review aimed to re-investigate helminth therapy for IBD and identify new evidence that might allow for a firmer conclusion on the concept of using helminths in UC and CD management.

## Methods

Systematic searches were conducted to identify studies that would be suitable for this review. PubMed, Embase, Medline and the Cochrane Central Register of Control Trials were used to carry out searches, and searches were conducted using MeSH headings including helminth, helminth therapy, IBD, UC and CD. Reference lists of studies that met the inclusion criteria were also searched to identify any studies that may have been missed by the database searches. The previous systematic review looking at a similar question regarding helminth therapy and IBD (Garg *et al*., 2014) was also examined.

In terms of developing the research question, the PEO (population, exposure, outcome) framework (Khan *et al*., 2003) was used. A summary of this is outlined in Table 1.

**Table 1.** Development of the review question

Population	Adult humans Professionally diagnosed CD or UC
Exposure	Exposure to helminth therapy
Outcome	Change in disease condition (positive or negative), measured by disease indexes, haematological changes and/or observed mucosal changes by endoscopy. Reports of AEs to determine tolerability and safety.

An overview of the inclusion and exclusion criteria for the search results of this review can be found in Table 2. There was no exclusion based on the sex or ethnicity of the participants, and there was no limitation on the date the studies were published as the aim of this review was to provide a comprehensive summary of any available evidence.

**Table 2.** Inclusion and exclusion criteria

Inclusion	Exclusion
Adult humans (18 years + ) Professionally diagnosed CD or UC English language Primary literature – RCTs, double-blind trials, single-blind trials, open-label studies.	Children (0–17 years) Animal model studies Cell studies Non-English publications Case reports Secondary literature – E.g. systematic reviews, literature reviews Letters, conference reports

**Fig. 1.**
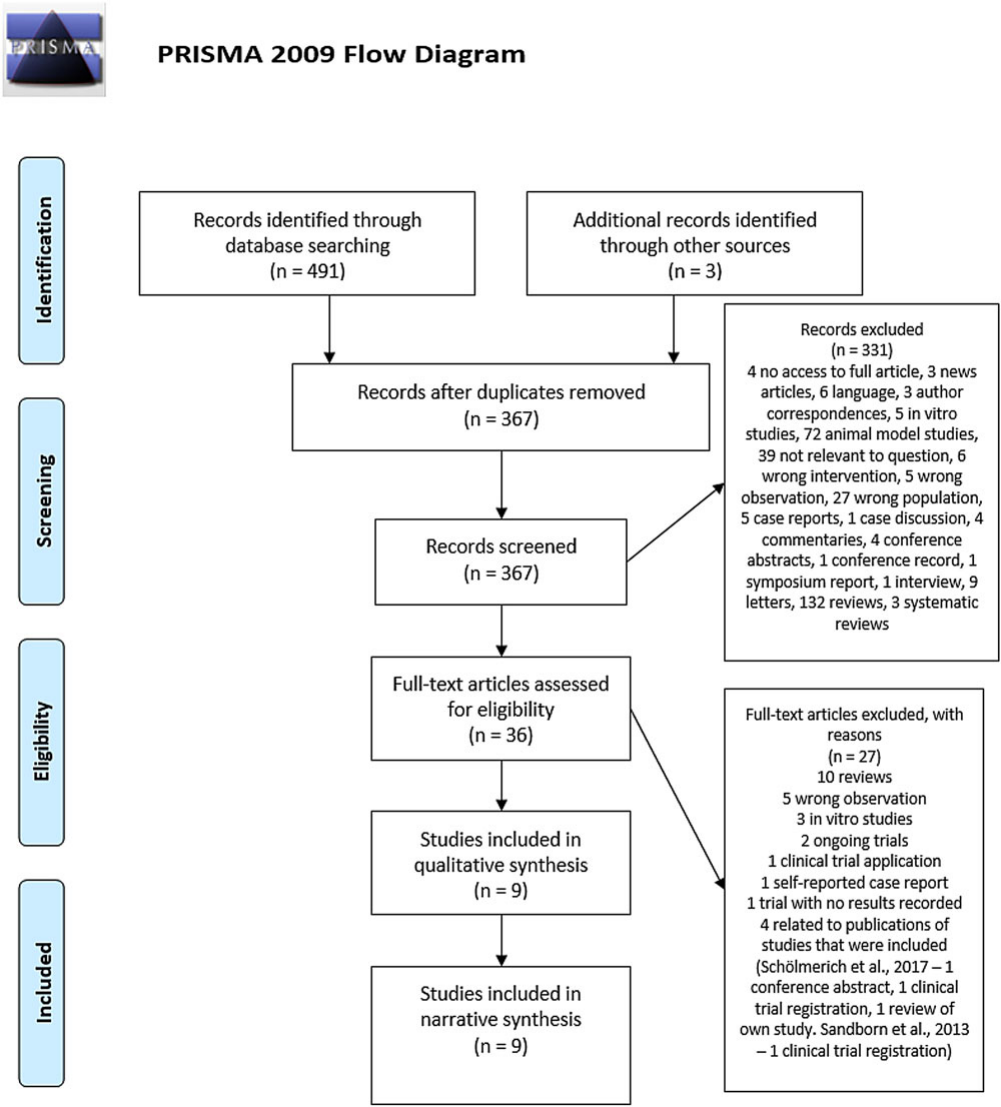
PRISMA flow diagram. The PRISMA diagram (Moher *et al*., 2009) shows the steps of the search and selection process detailed in the ‘Methods’ section of this review.

Study selection was conducted in two phases. In the first phase, titles and abstracts were screened. In the second phase, full texts were assessed to determine their eligibility for inclusion. The stages in the PRISMA flow diagram (Fig. 1) (Moher *et al*., 2009) were followed to identify and screen the results of the initial search and to identify studies eligible for inclusion in the review.

Data extraction was carried out using a data extraction form which was designed using the Joanna Briggs Institute (JBI) guidance on data extraction (Tufanaru *et al*., 2020) and the Cochrane Handbook for Systematic Reviews of Interventions (Li *et al*., 2019). The CASP (Critical Appraisals Skills Programme) randomized control trial (RCT) checklist (Critical Appraisal Skills Programme, 2018) was used to critically appraise the studies that were RCTs, and the methodological index for non-randomised studies (MINORS) assessment tool (Slim *et al*., 2003) was used to appraise the open-label studies.

Due to there being quite a lot of heterogeneity between some of the studies (i.e. varying study designs, different treatments used and differing methods of measuring the outcomes) a meta-analysis was not suitable. As such, a narrative synthesis was used to consistently summarize all the findings of the studies.

This was conducted by one individual under the supervision and guidance of another experienced in completing systematic reviews.

## Results

### Search results and study characteristics

The database searches identified 491 records. An additional three studies were identified during a search of the background literature on the subject. After the removal of duplicates, 367 remained for the title and abstract screening. Following this, 331 studies were excluded leaving 36 studies for the phase 2 full-text screening. Screening of the full texts excluded 27 studies, leaving nine that were eligible for inclusion in the review (Summers *et al*., 2003, 2005*a*, 2005*b*; Croese *et al*., 2006; Sandborn *et al*., 2013; NCT01576471, 2017; NCT01953354, 2017; Schölmerich *et al*., 2017; Capron *et al*., 2019). The results of the screening and exclusions/inclusions were documented using the PRISMA flow diagram (Fig. 1) (Moher *et al*., 2009).

Table 3 provides a full summary of the eligible studies in the review. Eight of the eligible studies used live helminth ova/ larvae. Of these, seven used *T. suis* ova with the dose ranging from *250 T. suis* ova (TSO) *to 7500 TSO,* and one used *N. americanus larvae*. One study used P28 glutathione-*S*-transferase (P28GST) (Capron *et al*., 2019), a protein derived from Schistosoma. Of the nine studies that were included six involved patients with CD (Summers *et al*., 2005*a*; Croese *et al*., 2006; Sandborn *et al*., 2013; NCT01576471, 2017; Schölmerich *et al*., 2017; Capron *et al*., 2019), two involved patients with UC (Summers *et al*., 2005*b*; NCT01953354, 2017), and one involved patients with both (Summers *et al*., 2003). Five were double-blind RCTs (Summers *et al*., 2005*b*; Sandborn *et al*., 2013; NCT01576471, 2017; NCT01953354, 2017; Schölmerich *et al*., 2017), and four were open label studies (Summers *et al*., 2003, 2005*a*; Croese *et al*., 2006; Capron *et al*., 2019). Two of the RCTs were the published results of clinical trials, not fully written articles (NCT01576471, 2017; NCT01953354, 2017). Eight studies looked at the efficacy of the treatment and made at least some comment on the safety (Summers *et al*., 2003, 2005*a*, 2005*b*; Croese *et al*., 2006; NCT01576471, 2017; NCT01953354, 2017; Schölmerich *et al*., 2017; Capron *et al*., 2019), and one looked solely at the safety (Sandborn *et al*., 2013).

**Table 3.** Summary of studies

Authors, (year), location	Title	Population	Study design	No. participants	Age range of participants (years)	Study overview	Primary outcomes observed	Secondary outcomes observed
Capron *et al*. (2019), France	Safety of P28GST, a Protein Derived from a Schistosome helminth parasite, in patients with CD: a pilot study (ACROHNEM)	Patients with CD	Open-label	10 (however two discontinued before the first injection)	22–44	P28GST was produced as a recombinant protein from *Saccharomyces cerevisiae* yeast cultures suspended in 1 mL of apyrogenic, sterile 0.2% alhydrogel solution, and administered with the adjuvant alum. Three Injections of 100 *μ*g of P28GST were given (1 per month) with a further 9-month follow-up period after the final injection.	1) Adverse events (AEs) AEs were classified into the following subgroups: − AEs of special interest (AESIs) − Other AEs − Serious AEs (SAEs) SAEs that were suspected as being related to the treatment but never described previously were called suspected, unexpected serious adverse reactions (SUSARs).	1) Change in CDAI 2) Serum calprotectin 3) Faecal calprotectin 4) Serum C-reactive protein (CRP) 5) Specific anti P28GST IgG, IgA, and IgE antibodies were measured at baseline, 3 and 12 months
Croese *et al*. (2006), Australia	A proof of concept study establishing *Necator americanus* in Crohn's patients and reservoir donors	Patients with CD	Open-label	9	21–55	Five participants were inoculated with *n* (25–50) 3rd stage *N. americanus* larvae and observed for 20 weeks. Following this, four additional participants were inoculated with n (50–100) 3rd stage *N. americanus* larvae, and the original cohort were reinoculated between week 27 to week 30 with their original inoculum. The total duration of the study was 45 weeks.	1) Changes in CDAI 2) IBD questionnaire 3) Blood eosinophil levels 4) Haemoglobin levels	1) AEs
NCT01576471 Study Director: Nova Silver (2017), USA	Efficacy and safety of TSO as compared to placebo	Patients with moderate to severely active CD	RCT	250	18–65	TSO 7500 every 2 weeks for 10 weeks (up to 6 doses total) or placebo every 2 weeks for 10 weeks (up to 6 doses total)	1) Efficacy of TSO defined as a reduction in the CDAI of ≥100 from participant's baseline 2) Reporting of any AEs (SAE = serious adverse event N-SAE = non-serious adverse event)	N/A
NCT01953354 Study Chairs: Hanauer and Jabri (2017), USA	TSO Treatment in left-sided UC	Patients with left-sided UC	RCT	16	18–70	Six doses of TSO 7500 orally over a 10-week period at 2-week intervals or six doses of TSO placebo orally over a 10-week period at 2-week intervals. Final follow-up was carried out at week 12.	1) Percentage of participants who achieved clinical response at week 12 (defined as a Mayo Score reduction of at least 3 points and at least a reduction of 30% from baseline, along with a decrease from the baseline rectal bleeding subscore of >1 point or a subscore of 0–1) 2) AEs	1) Percentage of participants who achieved remission at week 12 (defined as Mayo score of 1 with absence of rectal bleeding and endoscopy score of 0 or 1) 2) Percentage of participants with healed colonic mucosa at week 12 (defined as Mayo score of 0 or 1) 3) Percentage of participants with modified clinical response (defined as reduction in modified Mayo score (i.e. no endoscopy component) of at least 2 points from baseline) 4) Time to modified clinical response (number of days taken to reach a modified clinical response) 5) Percentage of participants with colonoscopic evidence of visible worms 6) Percentage of participants with increased diarrhoea (defined as an increase in the Mayo Score Stool Frequency Score by at least 1 point 7) Percentage of participants with an increase in concurrent UC medications or new rescue medications added
Sandborn *et al*. (2013), USA	Randomized clinical trial: The safety and tolerability of TSO in patients with CD	Patients with CD	RCT	36	18–55	The treatment groups in this study were TSO 500 *n* = 9, TSO 2500 *n* = 9, TSO 5000 *n* = 9, placebo *n* = 9. Following inoculation participants received a follow-up 2 weeks later. After the initial follow-up at 2 weeks participants were contacted by telephone at 1, 2 and 6 months after the study to discuss any further changes in symptoms.	1) Reporting of any AEs or symptoms	1) Examination of stool sample for presence of parasites and ova.
Schölmerich *et al*. (2017), Austria, Czech Republic, Denmark, Germany and Switzerland	A randomized, double-blind, placebo-controlled trial of TSO in Active CD	Patients with mild-moderately active CD	RCT	252	18–75	Six fortnightly doses of either 250, 2500, or 7500 TSO/15 mL suspension/ day, or 15 mL placebo solution/day, with eight visits at weeks 0, 1, 2, 4, 6, 8, 10, and 12, and a follow up visit 4 weeks after the final dose.	1) The proportion of patients that achieve clinical remission (defined as a CDAI <150)	1) The course of clinical remission. 2) The proportion of participants who achieved a reduction in the CDAI of >100 compared to their baseline. 3) Changes in inflammatory markers (CRP, faecal calprotectin). 4) Mucosal healing, observed by optional ileocolonoscopy at baseline and end of trial. The Simple Endoscopic Score for CD was then used to rate the endoscopic findings. 5) Physicians' Global Assessment. 6) Change in blood eosinophilia count, eosinophil derived neurotoxin (EDN), and E/S antigen from baseline. 7) Adverse reactions to treatment
Summers *et al*. (2005*b*), USA	*Trichuris suis* therapy for active UC: A randomized controlled trial	Patients with active UC (defined as UCDAI of ≥4)	RCT	54	18–72	2500 TSO or placebo at 2 weeks intervals for 12 weeks.	1) Changes in the UCDAI and Simple Index. Defining an improvement in UCDAI as ≥4. Remission defined as UCDAI of ≤2.	1) Haematological, renal and hepatic profile done at entry, week 6, and week 12. 2) Examination of stool samples for presence of ova or parasites. 3) Reporting of any AEs.
Summers *et al*. (2003), USA	*Trichuris suis* seems to be safe and possibly effective in the treatment of IBD	Patients with CD and UC	Open-label	7 (4 with CD and 3 with UC)	32–65	Part 1: Single dose of 2500 TSO with follow-up every 2 weeks for 12 weeks following the administration. Part 2: (investigating multiple doses), additional doses of 2500 TSO every 3 weeks for ≥28 weeks.	1) Looking for efficacy response. Primary criteria for determining efficacy included the IBDQ, the CDAI for the CD participants and SCCAI for UC participants. 2) AEs	N/A
Summers *et al*. (2005*a*), USA	*Trichuris suis* therapy in CD	Patients with active CD (defined as CDAI ≥220, with eligibility criteria of 220–450)	Open-label	29	18–72	2500 TSO every 3 weeks for 24 weeks	1) Looking for either response or remission. Disease activity was monitored using the CDAI. Remission was defined as a decrease in the CDAI to <150. Response was defined as a decrease in the CDAI of >100.	1) AEs 2) Haematological measurements (FBC, blood urea, nitrogen, creatinine, alanine aminotransferase, aspartate aminotransferase and alkaline phosphatase)

The studies were all published between 2003 and 2019. Six were conducted in the USA (Summers *et al*., 2003, 2005*a*, 2005*b*; Sandborn *et al*., 2013; NCT01576471, 2017; NCT01953354, 2017), one was in Australia (Croese *et al*., 2006), and two were in Europe (Schölmerich *et al*., 2017; Capron *et al*., 2019).

The number of participants taking part in the studies varied between seven participants in the smallest study (Summers *et al*., 2003) and 252 participants in the largest (Schölmerich *et al*., 2017). The age range across all of the studies was 18–75 years, and all of the studies included a mix of male and female participants. None of the studies appeared to intentionally discriminate between ethnicities however the majority of participants were white/ Caucasian.

### Quality appraisal of the studies

The five RCT's were appraised using the CASP RCT checklist and the MINORS assessment tool was used to appraise the four open-label studies. Table 4 summarizes the methodological flaws identified in the studies. These include areas of potential bias such as whether studies used blinding and control groups, and if the study methodology was explicit.

**Table 4.** Summary of methodological flaws in the studies

Methodological flaw	Study
Inclusion/exclusion criteria – No inclusion/exclusion criteria reported	Croese *et al*. (2006)
Demographics – Not reported/ poorly reported	Croese *et al*. (2006)
Control groups – No control group	Croese *et al*. (2006), Summers *et al*. (2003), Summers *et al*. (2005*a*) (open-label study), Capron *et al*. (2019)
Groups not well matched	Sandborn *et al*. (2013), NCT01953354 (2017)
Blinding – Complete lack of blinding	Croese *et al*. (2006), Summers *et al*. (2003), Summers *et al*. (2005*a*) (open-label study), Capron *et al*. (2019)
Methodology not clear/source of final data not clear	Croese *et al*. (2006)
Statistical analysis – Lack of statistical analysis – Test type not clear	− NCT01576471 (2017), Summers *et al*. (2003). − Sandborn *et al*. (2013),
Sample size of study too small	Croese *et al*. (2006), Summers *et al*. (2003), Summers *et al*. (2005*a*) (open label study), Capron *et al*. (2019), NCT01953354 (2017), Summers *et al*. (2005*b*) (RCT).

### Summary of study findings

To answer the research question of this systematic review the results of the studies are categorized into those regarding efficacy and safety.

#### Efficacy of treatment

Eight of the nine studies assessed the efficacy of a helminth therapy (Summers *et al*., 2003, 2005*a*, 2005*b*; Croese *et al*., 2006; NCT01576471, 2017; NCT01953354, 2017; Schölmerich *et al*., 2017; Capron *et al*., 2019). Of these, six used *Trichuris suis* ova (TSO) as the treatment (Summers *et al*., 2003, 2005*a*, 2005*b*; NCT01576471, 2017; NCT01953354, 2017; Schölmerich *et al*., 2017), one used *Necator americanus* larvae (Croese *et al*., 2006), and one used P28GST (Capron *et al*., 2019). Five of the studies were looking at patients with CD (Summers *et al*., 2005*a*; Croese *et al*., 2006; NCT01576471, 2017; Schölmerich *et al*., 2017; Capron *et al*., 2019), two looked at patients with UC (Summers *et al*., 2005*b*; NCT01953354, 2017), and one looked at both (Summers *et al*., 2003).

Five of the eight concluded that helminth therapy seemed to be effective in patients with CD or UC (Summers *et al*., 2003, 2005*a*, 2005*b*; Croese *et al*., 2006; Capron *et al*., 2019). This was mostly measured through improvement in disease activity indexes with some studies investigating additional outcomes. These disease activity indices included the CD activity index (CDAI), the UC disease activity index (UCDAI), the simple clinical colitis activity index (SCCAI) and the IBD quality of life questionnaire (IBDQ). Only one of these studies was an RCT (Summers *et al*., 2005*b*), and as such had control for comparison, but all the studies showed a statistically significant improvement (where statistical analysis was available) (Summers *et al*., 2003, 2005*a*, 2005*b*; Croese *et al*., 2006; Capron *et al*., 2019). An additional finding in one of the studies was that maintenance doses were required to sustain the effect of the TSO treatment (Summers *et al*., 2003). This study involved the use of an initial single dose of TSO with 12 weeks of monitoring, followed by an extended trial in which some participants received repeat doses every 3 weeks. The extended trial found that this prolonged the duration of clinical improvement in all four participants who took part. The results from the study using P28GST also indicated the need for repeat dosing, as at the end of the monitoring period (9 months after the final dose), there was no longer a significant difference between the baseline and post-treatment CDAI scores (Capron *et al*., 2019). This finding suggests that there is a natural clearance from the body in the case of both live and molecular treatment, and that the continued presence of the helminth/helminth derived product is needed to maintain the therapeutic effect.

Three of the eight studies concluded that treatment showed no advantage over placebo, all of which were RCTs looking at TSO treatment, and again used changes in disease activity indices (in this case CDAI and the Mayo score) as the main measure of efficacy (NCT01576471, 2017; NCT01953354, 2017; Schölmerich *et al*., 2017).

There was some evidence of colonic mucosal healing in one of the studies which seemed to show a notable difference between the treatment group and the placebo (66.7% showed mucosal healing in the treatment group and 16.7% in the placebo group). However, after statistical analysis, this was not found to be statistically significant (*P* = 0.242) (NCT01953354, 2017).

One study (Schölmerich *et al*., 2017) which investigated different doses of TSO, ranging from TSO 250 to TSO 7500, did suggest that higher doses may have greater efficacy. With clinical remission in this study defined as CDAI <150, of the TSO 250 group 38.7% achieved remission and in the TSO 7500 group 47.2% achieved remission. However, as this study did not find a statistically significant difference between the treatment groups and the placebo group this finding should not be over-interpreted.

Measurements of inflammatory markers in one study (serum calprotectin, faecal calprotectin and CRP) showed that levels did decrease following the therapy, but when analysed this was not found to be statistically significant (Capron *et al*., 2019). However, another study that also measured inflammatory markers (CRP and faecal calprotectin) found no clinically significant change took place between the start and end of the study (Schölmerich *et al*., 2017). It is possible that this may be because the treatments used were different between these two studies.

In the studies that investigated the levels of eosinophils and antibodies specific to the treatment, there was an observed increase (Croese *et al*., 2006; Schölmerich *et al*., 2017; Capron *et al*., 2019), which is consistent and expected with parasitic infection (Huang and Appleton, 2016), These findings suggest that the treatment did exert an effect on the host's immune system. However, when taken with other outcomes measured this was not found to have an effect on the rate of disease remission (Schölmerich *et al*., 2017). After the completion of the studies, the eosinophil levels decreased after a period of time following the last treatment dose (Croese *et al*., 2006; Capron *et al*., 2019).

#### Safety

All of the nine studies commented on adverse events (AEs) experienced during the course of the study.

There was no incidence of mortality in any of the studies. The incidence rate of serious AEs (SAEs) was generally low across all of the studies, while the rate of non-serious AEs (N-SAEs) was higher. It should be noted that in the studies with control groups the rate of SAEs and N-SAEs was relatively similar between the treatment group and the placebo group.

In studies that looked at haematological measures (i.e. full blood count, renal profile, hepatic profile) there were no significant changes observed (Summers *et al*., 2005*a*, 2005*b*; Croese *et al*., 2006), suggesting that the treatments did not have a negative physiological effect on these systems.

The overall conclusion of all the studies was that the treatments could be considered safe and well-tolerated in the treatment groups (Summers *et al*., 2003, 2005*a*, 2005*b*; Croese *et al*., 2006; Sandborn *et al*., 2013; NCT01576471, 2017; NCT01953354, 2017; Schölmerich *et al*., 2017; Capron *et al*., 2019).

## Discussion

Whilst this systematic review identified more studies for inclusion than the similar study conducted in 2014 (Garg *et al*., 2014), like the previous review, it still does not reach a conclusive answer in regard to using helminth therapy for CD and UC. Arguably more studies indicated that helminth treatments seemed to be efficacious (five out of eight studies). However, the three that did not find any benefit were all RCTs that had a placebo for comparison and by virtue of their methodology were less likely to be biased than the open-label studies (Burns *et al*., 2011). Therefore, it seems that there is an almost equal balance of evidence both for and against helminth therapy.

It should be noted though, that all studies concluded that helminth therapy seemed to be safe and tolerable, which gives some support towards using it. IBD can be somewhat varied in its presentation but some of the reported AEs were consistent with symptoms of IBD (particularly the GI symptoms (Seyedian *et al*., 2019) but also some extraintestinal symptoms (Levine and Burakoff, 2011)), meaning a number of the AEs could have been the result of the disease rather than the treatment. This is supported by the RCT studies which showed a similar number of AEs in the placebo group to the treatment groups (Sandborn *et al*., 2013; NCT01576471, 2017).

As all the studies concluded that the use of helminth therapy was safe and tolerable the need for further research in this area is indicated. An important part of the studies in this review was identifying a helminth/helminth derived product that would be well tolerated in participants and ideally cause minimal symptoms. With regard to the live helminths used in the studies, *Trichuris suis* does not typically infect humans and exists naturally as a porcine parasite (Cutillas *et al*., 2009). Though not the usual host of *T. suis*, cases of cross infections in humans have been documented in the past, but there is no known disease specifically associated with infections in humans (Beer, 1976). This fact may be the reason it is an appealing choice for helminth therapy. *Necator Americanus* is a more unusual choice, as it is a natural human parasite and can produce symptomatic disease (Hotez *et al*., 2005), but the study that investigated it (Croese *et al*., 2006) did so with the rationale that exploiting its long lifespan (3–5 years) (Roberts *et al*., 2008) would be beneficial as it may reduce the need for repeated dosing.

### Implications for practice

The results of this review do not provide any evidence to suggest that helminth therapy is better than the current recommended treatments for IBD or that any changes should be made to current practice. This is consistent with existing guidelines which do not presently suggest helminth therapy as a treatment option (Lamb *et al*., 2019). However, as some of the studies demonstrate a degree of efficacy, there is the potential for it to be offered in the future as an alternative therapy for patients who are not responding to current recommended treatments. Whilst the reported efficacy may be the result of a placebo effect, it could be beneficial to a patient if they felt symptomatic relief. All the studies concluded that helminth therapy could be considered safe, so there is nothing currently to suggest that this could not be attempted. In the future, studies comparing the efficacy of helminth therapy to the current standard treatments (e.g. steroids), or investigating them as a combination therapy, may be of interest.

There may be an issue with patient compliance if helminth therapy did become an available treatment option as not all patients may be comfortable with the idea of being infected with live parasites. Although, it should be noted that there have been cases of patients intentionally infecting themselves with helminths in an attempt to self-medicate their condition (Ahrens, 2016; Meluban, 2019; Nelson *et al*., 2019). The fact that the studies included in this review managed to recruit voluntary participants to trial helminth therapy should also be considered, as it suggests a number of IBD sufferers are willing to try this treatment. Potentially helminth derived products, such as P28GST (Capron *et al*., 2019), may have more success in terms of appeal.

### Strengths

This review provided a comprehensive search of four databases, with an examination of reference lists and a previously published systematic review of a similar topic (Garg *et al*., 2014). Only relevant studies were included and the included studies were critically appraised with appraisal tools appropriate for the design of each study.

### Limitations

This review was limited by the fact that it only included studies that were available in the English language. This was due to the authors being solely English speakers, and as this study received no funding no translators could be hired. However, it should be noted that the screening process only excluded 6 studies out of a total of 367 due to a language barrier. Therefore, it is possible that relevant studies published in other countries may have been omitted resulting in a potential bias in this review, but, if so, the number is low.

A limitation within some of the studies themselves is the way in which efficacy was measured. There is some evidence suggesting that the CDAI, UCDAI and the Mayo score may not be the most reliable way of tracking disease activity in relation to the assessment of treatment and that they don't always correlate well with endoscopic assessment, inflammation and mucosal healing (Filik *et al*., 2006; Morris *et al*., 2013; Mihai *et al*., 2018). Whilst all are historically validated tools, they represent more of a view of the patient's subjective well-being than the actual level of mucosal inflammation seen in the disease (Mihai *et al*., 2018). There is some belief that the inclusion of inflammatory biomarkers (such as CRP, ESR and faecal calprotectin) might provide a better assessment of disease activity (Morris *et al*., 2013; Rogler and Biedermann, 2015; Mihai *et al*., 2018), which two of the studies in this review did additionally investigate (Schölmerich *et al*., 2017; Capron *et al*., 2019). This suggests that there are additional outcomes that were not considered by all the studies, and that the measure of efficacy used may not always have been truly accurate. Furthermore, many of the disease indices used in the studies in this review have self-reported elements, and due to differences in the judgement of individuals, this could create some variation or bias in reporting.

Another limitation of this review was that database searches and data extraction was only carried out by one individual. As such, there is potential for consistent biases or mistakes throughout the review without a second reviewer to minimize the risk of this with a differing opinion. It should be noted though that this systematic review was completed under the supervision and guidance of another individual with experience in carrying out systematic reviews.

## Conclusion

The results of this systematic review suggest that helminth therapy is safe for use and may potentially have some efficacy, however further research (particularly with control groups) is needed to provide stronger evidence for this. Future studies should also consider the use of inflammatory markers as a means of more reliably monitoring disease activity in response to treatment. As it stands, it is difficult to conclusively rule out a placebo effect as the cause of any efficacy seen following treatment. Additional examination of dose strengths in clinical trials could be of interest as only three studies explored this, and of these only two looked at the efficacy of treatment. This also applies to dosing regimens, as this was not particularly explored in the studies in this review. It is possible that products derived from helminths could be more tailored for therapeutic use than living organisms, but as it stands, based on the results of one study, it is not possible to recommend advantages to molecular therapy over the live parasite without further research. P28GST did show promise as an efficacious and safe treatment for CD, so there does appear to be potential in this.
